# Event-Related Potentials to an English/Spanish Syllabic Contrast in Mexican 10–13-Month-Old Infants

**DOI:** 10.5402/2012/702986

**Published:** 2012-02-29

**Authors:** Maritza Rivera-Gaxiola, Adrian Garcia-Sierra, Lourdes Lara-Ayala, Cesar Cadena, Donna Jackson-Maldonado, Patricia K. Kuhl

**Affiliations:** ^1^Institute for Learning and Brain Sciences, University of Washington, Seattle, WA 98195-7988, USA; ^2^Facultad de Lenguas y Letras, Universidad Autonoma de Queretaro, 76140 Queretaro, QRO, Mexico; ^3^Department of Speech and Hearing Sciences, University of Washington, Seattle, WA 98195, USA

## Abstract

We report brain electrophysiological responses from 10- to 13-month-old Mexican infants while listening to native and foreign CV-syllable contrasts differing in Voice Onset Time (VOT). All infants showed normal auditory event-related potential (ERP) components. Our analyses showed ERP evidence that Mexican infants are capable of discriminating their native sounds as well as the acoustically salient (aspiration) foreign contrast. The study showed that experience with native language influences VOT perception in Spanish learning infants. The acoustic salience of aspiration is perceived by both Spanish and English learning infants, but exposure provides additional phonetic status to this native-language feature for English learning infants. The effects of early experience and neural commitment as well as the impact of acoustic salience are further discussed.

## 1. Introduction

There is a robust corpus of behavioral studies showing that, at a young age, infants discriminate phonetic contrasts from different languages of the world (e.g., [1–4]). The capacity of infants to acquire linguistic information from their native language is well established, but the mechanisms—neural or other—underlying the plasticity of the infant brain as well as the impact of linguistic exposure on acquisition are not well understood. In fact, the behavioral literature documents developmental changes for infants in various linguistic environments, but to our knowledge no data have been reported regarding cross-linguistic speech perception and its neural correlates in normally developing Spanish learning Mexican infants, more specifically comparing their neural responses to those of infants of the same age using the same phonetic stimuli, but learning English. Our goal in the present study was to enhance our understanding of how exposure to the phonetic units of a particular language, as well as acoustic salience, differentially affects the type and timing of neural responses to phonetic units early in development and how these relate to neural plasticity and neural commitment. 

It is accepted that infants aged 6–8 months discriminate most native and foreign consonant differences at a similar behavioral level [5–7]. For vowels, the effects of language experience are already present at this age [8]. By 10–12 months of age linguistic experience affects the perception of consonants; there is a decline in the ability to discriminate foreign consonant contrasts [5–7, 9–14], and more recent reports have also demonstrated that perception of native-language phonetic consonants increases significantly at these ages [7, 15]. More specific to the present study, Eilers and her colleagues [16] reported in a cross-linguistic study that Spanish-learning 6–8-month-old infants were capable of discriminating both an English and a Spanish voicing contrast, whilst infants the same age but acquiring English could only overtly respond to the English contrast. Eilers and collaborators suggested that specific auditory experience was important in the discrimination of the Spanish contrast, but that the English contrast contains other acoustic information (aspiration) that makes it easier to discriminate, even in the absence of specific exposure. In other words, Eilers et al. [16] argued that the English contrast was relatively unaffected by linguistic history, because of it acoustic salience [16]. However, the study was criticized based on methodological issues [17].

Both the discriminatory capacities as well as the enhancement in native-language phonetic discrimination have been demonstrated using neural responses [18–24]. Neural responses, such as the recording of event-related potentials (ERPs), provide a continuous record of brain activity during the time period in which the cognitive processes under investigation actually occur. ERPs are part of the voltage changes within the electroencephalogram (EEG) that are related to the brain's response to specific events. In other words, ERPs are the brain voltage changes time-locked to the presentation of some external (presentation of a stimulus, e.g.) or internal (decision making, e.g.) event. The waveforms' deflections are typically classified according to polarity (Positive or Negative), sequence (first positive peak, etc.), and latency (time window in ms where a peak occurs, e.g.). Different ERP patterns recorded during speech processing may reflect different levels of representation or information processing [25].

Of particular interest to the present report are the findings of Rivera-Gaxiola and collaborators [19] describing auditory brain potentials to native and foreign contrasts in a longitudinal study where American infants learning English were tested at 7 and 11 months of age. In that study, using CV-syllables differing in VOT, ERP group differences at 7 and 11 months of age mirrored the behavioral literature regarding perception of native and foreign contrasts [5–7, 12]. More specifically, at 7 months of age, infants showed evidence of neural discrimination to both Spanish and English contrasts, whilst at 11 months of age, ERP group data showed no evidence of discrimination to the foreign (Spanish) contrast and an enhancement of neural discrimination to the native (English) contrast. The authors observed that neural discrimination at the group level was represented by ERP amplitude differences in a negativity occurring between 250 and 550 ms after syllable onset (henceforth N250–550) but they also described differences over an earlier, positive-going deflection occurring between 150 and 250 ms after stimulus onset when infants' ERPs were evaluated individually. While one group of infants showed a larger P150–250 in response to both contrasts at 7 months, the other group of infants showed a larger N250–550. When evaluated at 11 months of age, the group that responded over the P150–250 time window at 7 months of age remained the same for the foreign contrast but changed to an N250–550 response to the native contrast. On the other hand, infants in the N250–550 responding group at 7 months remained N250–550 responders to both types of contrasts at 11 months. The authors concluded that ERP group data may underestimate individual discriminatory capacities and that the brain remains capable of discriminating foreign contrasts at 11 months of age; that is, there is no absolute “loss” in sensitivity to the foreign contrast, as also shown by the adult behavioral data [13, 26], and later buttressed by adult electrophysiological data [27], and other reports on infants [6, 7]. Also, differences in polarity in ERPs to speech stimuli during the first year of life have been widely documented [18, 28–44], however, there is no current consensus about what these different timing and polarity effects mean.

In a second study, Rivera-Gaxiola and collaborators [20] replicated the patterns described earlier using the same methods on a larger sample of 11-month-old monolingual American infants. In this second study, the authors reported that responding over the P150–250 time window (P-response) or over the N250–550 time window (N-response) to the foreign contrast at 11 months of age predicted vocabulary production at 18, 22, 25, 27, and 30 months of age. Infants displaying a “P-response” to the foreign contrast produced significantly more words than infants showing an “N-response” at the same ages. The authors conclude that showing a “P-response” to the foreign contrast and an “N-response” to the native contrast signifies more commitment to their native language than displaying an “N-response” to both contrasts (see [24, 45] for additional discussion). Furthermore, in a scalp distribution analyses considering 7-, 11-, 15-, and 20-month-old infants, corroboration was obtained by showing that the P150–250 and the N250–550 components also differ in scalp distribution [21]. Dehaene-Lambertz and Gliga [46] have also reported that different ERP components with different topographies can signal different types of underlying neural processing (see [47] for a review). In other words, in our studies, displaying an “N-response” to both contrasts means that the infant is not attending differentially to native language patterns and this in turn affects later language development. A recent study with bilingual infants learning Spanish and English showed that the strength of ERP response to either language predicted word production in that particular language 6 months later [43].

The goal of the present study was to replicate, extend and compare our previous developmental and cross-sectional findings that combined electrophysiological measures (namely, Event-Related Potentials) and a cross-language approach. Do Mexican infants learning Spanish show the same ERP differences observed in monolingual American infants? We hypothesized that 10–13-month-old infants learning Spanish in Mexico would show ERP discriminatory evidence for both the native and foreign contrast types: as a group, these infants would show early P150–250 differences to the foreign contrast because of its acoustic salience, and N250–550 differences to the Spanish contrast because it is native to them. Using the exact same stimuli, and following the exact same criteria as the Rivera-Gaxiola and collaborators' previous studies [19, 20], we also expected to encounter a subgroup of infants responding with amplitude differences over the P150–250 time window and another one responding with amplitude differences at the N250–550 time window to the foreign contrast.

## 2. Methods

### 2.1. Participants

Thirty five Mexican infants (20 boys/15 girls) aged 10–13 months (mean age = 11.5, range 10.10–13.5 months) were recruited by word of mouth in two different cities in Mexico. All infants were acquiring Spanish as their mother tongue and lived in monolingual households. All infants recruited were born at term (>39 weeks), with a normal birth weight (6–10 lbs.), and their mothers had normal pregnancies and deliveries. Infants were reportedly healthy at the time of recording. Parents signed UW ethics committee-approved consent forms and procedures were described in Spanish before testing. Mexican parents received forms in Spanish and were paid $15 dollars for their participation in the study.

Data from 18 of the Mexican infants (9 boys and 9 girls) were accepted for ERP analyses and are here reported. Data from the remaining infants recruited and recorded were eliminated due to a low number of artifact-free trials to average (total number of trials = 1000, with 800 standards and 200 deviants including native and foreign; minimum number of deviant trials to be accepted for ERP analyses = 80).

### 2.2. Stimuli

The stimuli used in the present study were identical to those used in previous investigations with English monolingual infants (see [19, 20] and Spanish/English bilingual infants [43]. In brief, CV syllables naturally produced by a Spanish/English bilingual female were used. The syllables differed in the relative onset of voicing versus the syllable burst, or voice-onset time (VOT), and included a phoneme that was common to both languages, an unaspirated alveolar voiceless /ta/ (heard as “da” by native English speakers and heard as “ta” by Spanish native speakers; VOT = 12 ms), a voicing-lead dental /da/ (heard as “da” by Spanish and English native speakers; VOT = −24 ms), and a voiceless aspirated alveolar English /t^h^a/ (heard as “ta” by English and Spanish speakers; VOT = 46 ms). Syllables were equal in duration (229.65 ± 0.3 ms), intensity, average root mean square power, and vowel color. The fundamental frequency was 180 Hz.

Behavioral discrimination of the experimental stimuli was tested using the active oddball paradigm in adult native speakers of English and in adult native speakers of Spanish. The repeated standard was the unaspirated alveolar voiceless sound common to both languages. Adult native English speakers scored 90% correct when the comparison was the English voiceless aspirated /t^h^a/ and 7% when the comparison was the Spanish voiced /da/. In contrast, native adult Spanish speakers scored 85% correct on the /t^h^a/ and 98% correct on the Spanish /da/. Native Spanish speakers reported that the English voiceless aspirated /t^h^a/ was a “strange” form of /ta/. Syllables were tested beforehand to ensure goodness of the English /da/ and /t^h^a/ in English speakers and goodness of the /ta/ and /da/ in Spanish speakers. The syllables selected were equally good: native English and native Spanish speakers rated their native syllables with “best” labels from a selection of “poor”, “fair”, “ambiguous”, “good”, and “best” options. Native Spanish speakers considered the aspirated English /t^h^a/ as “poor.” Head-turn behavioral studies in our laboratory show that 11-month-old American infants discriminate their native contrast (/ta/-/t^h^a/) at about 70 percent correct, well above chance, and the foreign /da/-/ta/ near chance at about 53% percent correct [48, 49].

### 2.3. Design

Participants were tested using the same double-oddball paradigm used in our studies with English monolingual infants and Spanish/English bilingual infants. The voiceless unaspirated sound (Spanish /ta/; English /da/) was used as the standard sound and it was presented 80% of the time. The remaining two syllables were the deviants (Spanish /da/ and English /ta/), each presented 10% of the time in a semirandom fashion: no consecutive deviants occurred and at least 3 standards occurred between deviants. The inter-stimulus interval (offset to onset silence) was 700 ms. One minute silences were inserted every *≈*2 minutes to allow for interaction with the infant. The syllables were played by two loudspeakers placed approximately 1 m in front of the child, at 69 dBA SPL. EEG from all participants was recorded while they were passively listening to the series of 1000 CV syllables.

### 2.4. Procedure

Infants were awake and sat on a high-chair placed inside a silent room or on the parent/guardian's lap. The parent or guardian sat next to/with the child. A research assistant was located in front of the infants and entertained them with silent toys. In addition, a silent video was presented on a TV monitor.

### 2.5. EEG Recording

The electroencephalogram (EEG) amplifier used was the isolated bioelectric amplifier system (SC-16/24 BA; SA Instrumentation San Diego, CA). The EEG was recorded using electrocaps (Electro Cap International) with preinserted tin Ag/AgCl electrodes and referenced to the left mastoid from Fp1, Fp2, F3, F4, C3, C4, P3, P4, F7, F8, T3, T4, Fz, Cz, and the active right mastoid of the 10/20 International system. The vertical electro-oculogram (VEOG) was recorded from 1 infra-orbital electrode placed on the infant's left upper cheek. The amplifier bandwidth was set between 0.1 and 40 Hz. All electrode impedances were kept homogeneous across sites and below 5 KΩ. Signals were amplified with a gain of 20000. EEG was sampled every 4 ms. EEG segments of 700 ms with a prestimulus baseline time of 100 ms were selected and averaged off-line to obtain the ERPs. Baseline correction was performed in relation to this prestimulus time. Further lowpass filtering was set at 15 Hz. Segments with large eye movements, blinks, or other artifacts were automatically eliminated (electrical activity exceeding ±150 *μ*V was considered artifact). ERP data were accepted for analyses when at least 80 artifact-free trials for each type of deviant and 80 of the standard could be obtained. All ERPs accepted showed clear auditory P-N complexes within the first 600 ms.

### 2.6. Data Analyses

Following Rivera-Gaxiola and collaborators [19], the waveforms obtained were explored within three time windows: N30–120 (negative deflection between 30 and 120 ms after stimulus onset), P150 and 250 (positive deflection between 150–250 ms after stimulus onset), and N250–550 (negative deflection 250–550 ms after stimulus onset). Peak amplitude values for standard preceding a deviant and deviant stimuli within each time window for each child and condition were obtained and used to calculate three independent three-way repeated-measures ANOVAs. The factors included were condition: native contrast (standard voiceless unaspirated versus deviant Spanish voiced /da/) and foreign contrast (standard voiceless unaspirated versus deviant voiceless aspirated /t^h^a/), lateral electrode position (left and right), and anterior-posterior location (frontal-polar, frontal, central, parietal, frontal-lateral, and temporal). For simplicity purposes, the speech contrast relating the standard voiceless unaspirated sound and the deviant Spanish voiced /da/ is referred to here as the *native contrast*. Also, the speech contrast relating the standard voiceless unaspirated sound and the deviant voiceless aspirated /t^h^a/ is referred to here as the *foreign contrast*.

## 3. Results

### 3.1. Group Results

Data from 18 participants (9 girls) could be included in the analyses (at least 80 artifact-free trials of each and all types of syllables). ERPs from all of these showed clear N30–120/P150–250/N250–550 complexes distributed over frontal, central, parietal, and temporal recording sites. The N30–120 was small but present, and not significantly different across conditions.

At the group level, normally developing 10–13-month-old Mexican infants learning Spanish displayed statistically significant amplitude differences between standard and deviant over the N250–500 time-window (N-response) for the native contrast, *F*(1,17) = 6.29  *P* = 0.02, partial *η* ^2^ = 0.270, and observed power = 0.658. The amplitude differences between standard and deviant for the foreign contrast over the N250–550 time window only approached significance, *F*(1,17) = 4.0  *P* = 0.06, partial *η* ^2^ = 0.191, and observed power = 0.471. However, as a group, these infants did show an important P150–250 peak amplitude difference (P-response) between standard and deviant for the foreign contrast [/ta/ versus /t^h^a/], *F*(1,17) = 9.636, *P* = 0.007, partial *η* ^2^ = 0.376, and observed power = 0.830, but not for the native contrast [/ta/ versus /da/], *F*(1,17) = 1.370, *P* = 0.259, partial *η* ^2^ = 0.079, and observed power = 0.196. 

**Figure 1:**
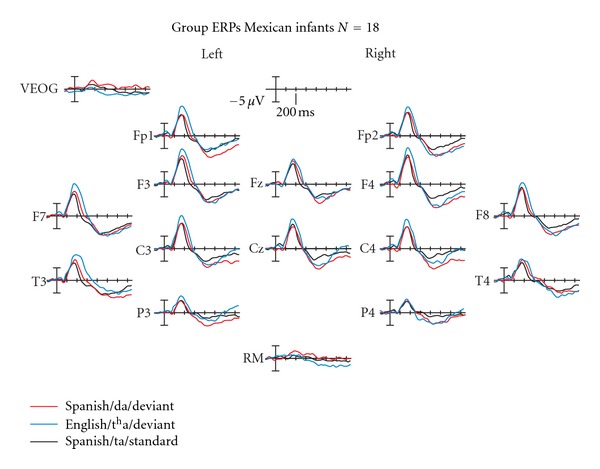
ERP pattern for the whole group (*N* = 18) to both the native and foreign contrasts. The group displays a significantly more negative N250–550 to their native deviant and a significantly more positive P150–250 to the foreign deviant. Positive is plotted up.

No left-right hemispheric differences were observed but there was an anterior-posterior main effect at the group level, with anterior amplitudes being larger than posterior ones, *P* < 0.05 at both P150–250 and N250–550 time windows for both conditions (Figure 1).

In order to obtain the same type of ERP information as in our previous studies, we followed the criteria of Rivera-Gaxiola and collaborators [19] and Rivera-Gaxiola and collaborators [20] and examined infants' ERP responses individually. Precisely, infants were subdivided into two groups according to the polarity and amplitude of the individual auditory ERP components observed in the foreign contrast condition. We have previously reported that the foreign contrast has important implications for later language development [20, 24]

As stated previously, all infants showed larger N250–550 amplitude differences for the native contrast /ta/ versus /da/ and no amplitude difference in the P150–250 time range. Therefore, the analyses given below are only for the foreign contrast.

### 3.2. Sub Group Results (Foreign Contrast [English /t^h^a/ Deviant])

In this sample of Mexican infants, we observed that 61% of the participants responded with larger peak amplitudes to the foreign /t^h^a/ deviant with respect to the standard over the P150–250 time-window  (*N* = 11, 6 girls), (see Table 1 for mean amplitude values over Cz for each type of syllable and time-window); 39% showed peak amplitude differences over the N250–550 only (*N* = 7 infants, 3 girls) (see Table 1 for mean amplitude values over Cz). The first group is the “P-response” subgroup and the second one the “N-response subgroup.”

#### 3.2.1. P-Response Subgroup Statistical Analyses

**Figure 2:**
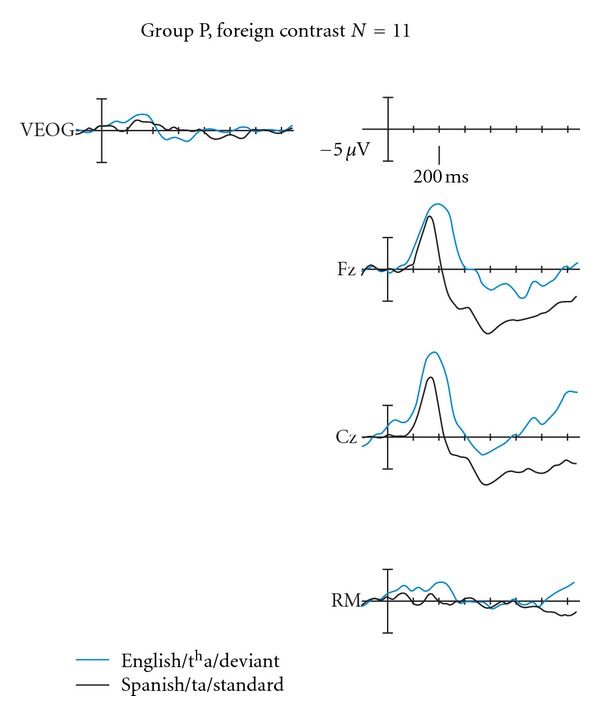
ERP pattern for Group P (*n* = 11) for the foreign contrast. These subjects respond over the P150–250 time window by showing larger amplitudes to the deviant /t^h^a/ with respect to the standard Spanish alveolar voiceless /ta/. Electrode locations Fz and Cz are shown. Positive is plotted up.

 For this subgroup of infants, the amplitude differences over the P150–250 region were highly significant, *F*(1,10) = 29.307, *P* = 0.001, partial *η* ^2^ = 0.746, and observed power = 0.988, but not significant over the N250–550 time window (*P* > 0.05) (Figure 2).

#### 3.2.2. N-Response Subgroup Statistical Analyses

**Figure 3:**
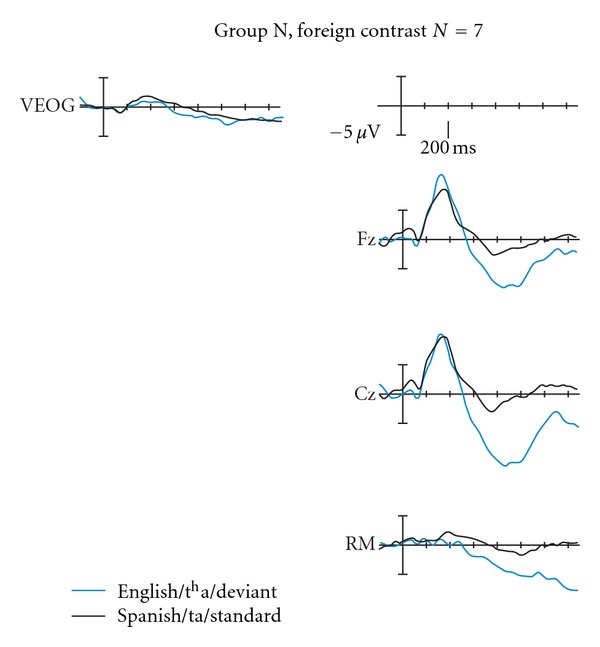
ERP pattern for Group N, foreign contrast (*n* = 7). These infants' responses to the foreign contrast approached significance over the N250–550 time window, with the deviant showing larger amplitudes. Electrode locations Fz and Cz are shown. Positive is plotted up.

 For these infants, we found that the differences only approached statistical significance, *F*(1,6) = 5.5, *P* = 0.058, partial *η* ^2^ = 0.476, and observed power = 0.5, which could be explained by the low number of infants (Figure 3).

Because the present study involved a double-oddball paradigm where one oddball is foreign to Spanish speaking infants and one is foreign to English learning infants, an important part of the research presented here was to compare the same paradigm and stimuli in both English and Spanish learning infants.  Table 2 presents a comparative table showing the responses obtained in the present Mexican sample and the ones previously reported for our American 11-month-old sample [19]; Table 2(a) shows group results, and Table 2(b) shows P- and N-responders' subgroups. At the group level, the results mirror the behavioral literature, but attention to individual variation supports the notion that the brain continues to process acoustic differences regardless of prior exposure. Both P- and N-responders show significant differences to their native contrast over the N250–550 time window.

## 4. Discussion and Conclusions

### 4.1. Group Results

In this study, we explored whether Mexican infants acquiring Spanish showed a similar pattern of ERP differences to native and nonnative phonetic contrasts that we observed previously when testing monolingual American infants using the exact same stimuli and analysis procedures [19–21]. We hypothesized that 10–13-month-old infants acquiring Spanish in Mexico would show ERP discriminatory evidence for both the native and foreign VOT contrast types, one reflecting the acoustic salience of the English lag contrast (seen in the early “P-response”) and the other reflecting the status of the Spanish VOT lead contrast as a phonemic contrast in the language (seen in the “N-response”). Our hypotheses were corroborated. Group results showed significant amplitude differences for monolingual Spanish infants' native /ta/-/da/ contrast over the N250–550 time window. Rivera-Gaxiola, Silva-Pereyra, and collaborators [20] reported that, at a group level, American 11-month-old infants also showed an “N-response” for their native /da/ versus /t^h^a/contrast. Moreover, the sample of Mexican infants also clearly responded to the foreign contrast /ta/ versus /t^h^a/, with the amplitudes of the P150–250 being significantly larger for the foreign deviant aspirated /t^h^a/, than for the standard /ta/. These results conformed to our hypotheses and also with the behavioral literature that reports that Spanish learning 6–12-month-old infants are able to respond to both the English and Spanish contrasts, regardless of phonemic status [50]. We hypothesize that the “P-response” observed in the majority (70%) of Mexican infants to the foreign contrast is reflecting acoustic discrimination of the English voicing contrast, which involves aspiration, an acoustically salient cue for all listeners, regardless of its phonetic status in their language. In other words, despite the fact that the aspirated /t^h^a/ does not have phonemic status in Spanish, inexperienced adults are able to perceive the aspiration in the syllable. The data presented here are consistent with our proposal that the “P-response” may reflect a less specialized (and more universal) form of detection of acoustic differences in speech contrasts.

It is also well known that there is a great deal of individual biological variability across infants, and this variability is meaningful in that it can predict future language abilities [15, 19–21, 24, 43, 45, 51]. It is therefore worthwhile to analyze individual ERPs. In the present work, we analyzed peak amplitude values of the P150–250 and the N250–550 components resulting from the presentation of a standard syllable and two different types of deviant syllables using the exact same method employed in our previous reports [19, 20]. We again observed that 10- to 13-month-old infants vary in the neural responses they display to a foreign contrast. When analyzing infants' responses to the foreign contrast, we observed a subgroup of infants who showed “P-responses” and a subgroup that displayed “N-responses.” 

### 4.2. Subgroup Results (Foreign Contrast)

 Our results show that eleven out of 18 infants displayed qualitative amplitude differences over the two time windows explored, though the only significant difference occurred over the P150–250 time window. The remaining 7 infants did not show “P-responses” and approached significance for the “N-response.” Rivera-Gaxiola and collaborators [19], showed that all of the American infants responded over the N250–550 time window for their native /da/ versus /t^h^a/ contrast at 11 months of age; some responded over the P150–250 time window and some over the N250–550 time window to the Spanish /ta/ versus /da/ foreign contrast. This pattern observed for the Mexican sample in the present study mirrors that seen in the American in the previous study, but for the opposite speech contrast. In short, across the two studies, we show that neural responses to native sounds at 11 months of age occur over the N250–550 time window, whereas neural discrimination to a foreign contrast can be reflected either as a “P-response” or as an “N-response.”

 Again, it is possible that the subgroup of infants with “N-responses” to both the native and the foreign contrasts are showing less native language neural commitment (NLNC, [52], as they reflect similar neural analyses to the two types of contrasts irrespective of the language status of those contrasts [19]. In the subset of “N-response” children, the strength of the “N-response” is smaller to the foreign than to the native contrast. This result is similar to Cheour and collaborators [18], who showed that a larger acoustical difference evoked a small MMN if the status of the vowel contrast was foreign. Displaying “N-responses” to a foreign contrast does not appear to be advantageous in monolingual language learning (see [24, 45] for discussion). In the Rivera-Gaxiola and collaborators [20] report, the American infants who showed the “P-response” to the foreign contrast later scored higher in vocabulary and sentence complexity (18 to 30 months of age) compared to their N-responding peers. Other behavioral [15, 48] and ERP data [24] also indicate a negative correlation between the capacity to discriminate foreign contrasts and concomitant word comprehension or later word production/comprehension.

Given the nature of the paradigm used, we cannot determine how the discriminatory responses observed in the populations studied to date are connected to attentional processes. We do not know what role the “P-response” plays in infants' behavioral discrimination responses to their native contrast at 6–8 months of age, but not at 10–12. This can only be explored using ERPs and behavioral methods with the same infants at the same time. Kuhl et al. [15, 24] examined both behavioral measures and ERP measures to native and foreign contrasts in the same infants at the same age but in separate sessions and showed significant correlations between an MMN-like difference waveform at 250–500 ms and performance in the behavioral task (Head-Turn conditioning). In both cases, better discrimination of the native contrast at 7.5 months predicted a more rapid advance in the acquisition of words and sentences, whereas better discrimination of the foreign contrast predicted a slower advance toward the mastery of words and sentences. Infants who showed an “N-response” to the foreign contrast in the present study, like those who showed a strong MMN-like difference waveform in Kuhl et al. [24] to the foreign contrast, may be infants whose native language neural commitment is not as advanced [52]. The presence of two types of responders may vary not only with age and exposure but also with the specific contrast involved. Cross-linguistic and developmental studies allow us to explore and understand which aspects of speech perception may hold for all languages or cultures, and which depend on experience. The fact that individual variation in the ERP responses predicts differences in later language development indicates that these differences are meaningful, and not simply “noise.”

In sum, across our studies, we have shown that the ERP responses from infants being raised in different cultures—English-learning, Spanish-learning, or both—are analogous and depend on the native or foreign status of a speech sound. For Spanish learners, the prevoiced /da/ is the native deviant and the aspirated /t^h^a/ is the foreign deviant, whereas for English learners the reverse is true, yet the infants responded similarly to their respective native and foreign deviants. Our ERP evidence reveals that the infant brain still responds to foreign sounds at 10–13 months of age, but that the neural responses to foreign sounds are weaker than to native sounds. Our results suggest that the brain retains a capacity to discriminate small differences in speech sounds regardless of prior exposure. Moreover, our results indicate that the time-course, polarity, and later language implications of the P150–250 and the N250–550 are different. These two electrophysiological components together reflect how the brain is shaped by exposure to a specific language. The “P-” and “N-responses” reported here are related to both acoustic salience and the phonetic status each deviant has for the infant depending on the language of her environment. We propose that because P-responders are allocating resources differentially depending on the phonetic status of the contrast involved, they are more committed to their native language and will advance more rapidly in language acquisition, while the N-responders are not distinguishing between what may be useful to attend to (native contrast) and what not (nonnative contrast) at this age, and therefore that their progression towards language may proceed at a different pace.

## Figures and Tables

**Table 1:** Mean amplitude values (in *μ*V) and standard deviation at electrode location Cz to the foreign contrast /ta/ versus /t^h^a/.

	P150–250 std /ta/	P150–250 dev /t^h^a/	N250–550 std /ta/	N250–550 dev /t^h^a/
*P* subgroup	*X* = 8.39 ± 5.01	*X* = 11.42 ± 5.84	*X* = −16.67 ± 12.34	*X* = −2.77 ± 9.95
*N* subgroup	*X* = 11.32 ± 10.76	*X* = 14.75 ± 15.78	*X* = −9.25 ± 4.67	*X* = −10.23 ± 5.68

**Table:** (a)

Group results Mexican *N* = 18/American *N* = 28	Contrast and phonetic status	ERP component P150–250 comparison	ERP component N250–550 comparison

Mexican infants	/ta/versus [t^h^a]	*F*(1,17) = 6.29, *P* = 0.02	N/S
(this study)	FOREIGN	partial *η* ^2^ = 0.270, OP = 0.830	
American infants	/ta/versus [t^h^a]	N/S	*F*(1,27) = 38.57, *P* = 0.001
[20]	NATIVE		partial *η* ^2^ = 0.588, OP = 1.0
Mexican infants	/ta/versus/da/	N/S	*F*(1,17) = 6.29, *P* = 0.02
(this study)	NATIVE		partial *η* ^2^ = 0.270, OP = 0.658
American infants	/ta/versus/da/	N/S	N/S
[20]	FOREIGN		

**Table:** (b)

Sub group results Mexican *N* = 18/American *N* = 24	Foreign contrast	P-responders	N-responders

Mexican infants (this study)	/ta/ versus [t^h^a]	*F*(1,10) = 29.307, *P *= 0.001**	*F*(1,6) = 5.5, *P* = 0.058
		partial *η* ^2^ = 0.746, OP = 0.988	partial *η* ^2^ = 0.476, OP = 0.5
American infants [20]	/ta/ versus /da/	*F*(1,12) = 26.41, *P *= 0.001**	*F*(1,10) = 16.44, *P *= 0.001**
		partial *η* ^2^ = 0.705, OP = 0.966	partial *η* ^2^ = 0.558
